# Self and Nature: Parental Socialization, Self-Esteem, and Environmental Values in Spanish Adolescents

**DOI:** 10.3390/ijerph17103732

**Published:** 2020-05-25

**Authors:** Pablo Queiroz, Oscar F. Garcia, Fernando Garcia, Juan J. Zacares, Cleonice Camino

**Affiliations:** 1 Faculty of Health Sciences at Trairi, Federal University of Rio Grande do Norte, 59200-000 Santa Cruz-Rio Grande do Norte, Brazil; pabloqueiroz@live.co.uk; 2 Department of Developmental and Educational Psychology, University of Valencia, 46010 Valencia, Spain; juan.j.zacares@uv.es; 3 Department of Methodology of the Behavioral Sciences, University of Valencia, 46010 Valencia, Spain; fernando.garcia@uv.es; 4 Departament of Psychology, Universidade Federal da Paraíba, 58033-455 João Pessoa-State of Paraíba, Brazil; cleocamino@yahoo.com.br

**Keywords:** parenting, parental socialization, parental warmth, parental strictness, self-esteem, environmental values, biospheric values, cultural context, Spain

## Abstract

Emergent research seriously questions the use of parental strictness as the best parenting strategy in all cultural contexts. Moreover, previous research on environmental socialization offers inconsistent findings about which specific parenting practices would be the most appropriate for environmental socialization. The present paper aims to examine parents’ contribution (i.e., authoritative, indulgent, authoritarian, and neglectful) to adolescents’ self-esteem and internalization of environmental values. Participants were 308 Spanish adolescents with 171 females (55.5%), between 12 and 17 years old. The four parenting styles were defined using measures of parental warmth and strictness. Self-esteem was captured with global and multidimensional measures. Internalization of environmental values was evaluated by measuring the priority given to biospheric values. Results revealed a consistent pattern between parenting styles and adolescent self-esteem and internalization of environmental values. Overall, adolescents from homes characterized by parental warmth (i.e., indulgent and authoritative) have higher self-esteem and greater internalization of environmental values than their counterparts. These findings clearly contrast with those obtained in other cultural contexts where parental strictness is essential in achieving well-adjusted children with optimal psychosocial development.

## 1. Introduction

Currently, environmental problems are taking place all over the world. The air we breathe is polluted, the water we drink stems from polluted rivers, and the land that feeds us is increasingly damaged in some way. Although the United Nations (2000) [1] warns us about the ecological crisis as one of the greatest threats to sustainable development, human beings still behave in ways that destroy the planet. For many years, researchers have examined the relationship between human actions and the environment. The socialization process is consistently identified as a crucial factor in the internalization of social values, including values associated with the conservation of nature [2]. An important goal of socialization is the transmission of values across generations, which is essential in the process of individual development and the functioning of society. Even in adolescence, when parents’ influence is reduced while other influences come from different socialization agents, such as peers or the mass media, the family plays a critical role as a protective or risk factor in adolescent functioning and competence [3,4,5,6,7].

The parenting styles approach is one of the most significant areas in the family socialization literature examining the relationship between parental actions and children and adolescents’ developmental outcomes. Traditionally, parenting styles have been studied focusing on two dimensions, warmth and strictness [6,8,9,10], defined as theoretically orthogonal (i.e., unrelated) constructs. Parental warmth is expressed in terms of responsiveness and acceptance, giving children and adolescents support and communicating with them. Scholars have used different labels to operationalize measures of parental warmth and acceptance, for instance, assurance [11], warmth [12], acceptance [13], love [14], responsiveness [15], or currently, acceptance/involvement [16,17]. Parental strictness is expressed in terms of supervision and maturity demands placed on the children. Other labels, similar to strictness, have also been used in the literature, such as control [12], domination [11], demandingness [15], or, currently, strictness/imposition [16,17]. The combination of these two main dimensions produces four parenting styles: authoritative (higher levels of warmth and strictness), indulgent (higher levels of warmth, but lower levels of strictness), authoritarian (lower levels of warmth, but higher levels of strictness), and neglectful (lower levels of warmth and strictness).

Many studies have examined the relationships between the four parenting styles and developmental outcomes in children and adolescents. However, there are inconsistent findings about which parental style is associated with the best developmental outcomes. Traditionally, studies show that the authoritative style (higher levels of both warmth and strictness) produces the best outcomes for children and adolescents’ psychosocial adjustment in middle-class European-American families [3,6,15,18]. Specifically, data suggest that children from authoritative families (higher levels of warmth and strictness) report higher levels of social and psychological capacities, better school achievement, and fewer behavioral problems [6,8,17]. In comparison with other styles, children and adolescents from neglectful households (lower levels of both warmth and strictness) have the worst scores on psychosocial adjustment. In an intermediate position between authoritative parenting (related to the best developmental outcomes) and neglectful parenting (related to the poorest developmental outcomes) are the authoritarian and indulgent styles. Regarding the authoritarian style, adolescents from these families report greater obedience and higher conformity with social norms, as well as less school misconduct, but relatively worse self-reliance and higher psychosocial and somatic distress. The indulgent style, in turn, has been related to some benefits for adolescents, such as a strong sense of self-confidence, but also with some psychosocial costs such as lower academic achievement, weak engagement in school, and more school misconduct. Nonetheless, empirical evidence does not support the idea of a universal protective role of the authoritative parenting style [19,20,21,22]. Several studies carried out in different ethnicities, environments, and cultural contexts show that other parental styles have a relationship with positive outcomes in children and adolescents. For example, in ethnic minority groups [16,23], in families with low socioeconomic status [24], and in Arab societies [25], the authoritarian parental style has been associated with better psychosocial adjustment in children and adolescents.

Additionally, a growing body of research, mostly conducted in Europe and Latin American countries, identifies benefits related to the indulgent parenting style (higher levels of warmth, but lower levels of strictness) on different indicators of psychosocial adjustment [26,27,28,29,30,31]. In fact, the indulgent parenting style is related to equal or even better outcomes than authoritative parenting, whereas the styles characterized by lower levels of parental warmth (i.e., authoritarian and neglectful) are associated with poor outcomes. Adolescents from indulgent households showed greater psychosocial maturity [32] and good performance in school. They developed academic competence based on self-regulated learning, achieving good school grades [21,33] and less academic stress and school misconduct [21,34]. In addition, the indulgent parenting style also provides ample benefits for personal competence in the realm of self-esteem. Adolescents from indulgent homes reported equal or better self-esteem than their peers from authoritative households, whereas adolescents raised by authoritarian and neglectful parents reported poor self-esteem [21,33,34]. Along the same lines, the indulgent parenting style also provides social competence in the realm of internalization of values. Adolescents from authoritative and indulgent households have higher levels of internalization of values such as self-transcendence (i.e., universalism and benevolence) and conservation (i.e., security, conformity, and tradition), whereas those from authoritarian and neglectful households are associated with the lowest internalization of values [7,35,36]. Components of parental warmth, such as involvement and reasoning with the children, might contribute to the priority given to social values by adolescents [37]. In addition, on other social values criteria, adolescents from indulgent and authoritative households gave higher priority to human rights principles than those from neglectful homes [38].

### Present Study

The present study examines the impact of parental socialization on self-esteem and the internalization of environmental values. Parental socialization is captured by the four-fold typology of parenting styles (i.e., indulgent, authoritative, authoritarian, and neglectful). Importantly, the environmental literature widely identifies the influence of family on the concern for the environment, conservation behaviors, and attitudes toward environmental protection in children through family processes such as the transmission of family environmental norms or the child’s observation and imitation of parental behaviors related to the environment [39,40,41,42]. For example, adolescents tend to report more energy-saving behaviors when their parents also have behaviors related to saving energy [43]. Nevertheless, less is known about the family as a context for pro-environmental socialization through parental practices of warmth and strictness, despite the well-documented impact of parental socialization on the broad spectrum of internalization of social values and prosocial behaviors.

Although empirical evidence about pro-environmental socialization is limited, scholars agree that general parenting practices (e.g., warmth, reasoning, granting autonomy, strictness, or monitoring) are related to adolescent pro-environmental behavior. However, research findings show important inconsistencies in explaining how parents engage in environmental socialization by applying environmental-specific parenting practices [41,44,45,46]. In particular, parental practices characterized by strictness are examined in environmental-specific socialization. For example, parents can insist that the child turn off lights to save electricity by withdrawing privileges due to noncompliance. Empirical evidence from a study [41] conducted in the United States, South Korea, and Israel did not find a relationship between environmental-specific parenting practices characterized by strictness and children’s environmental behavior (sustainable lifestyle, reducing consumption, and reducing impact). In contrast, findings from a study [45] with German adolescents and their parents revealed that environmental-specific parenting practices characterized by strictness (parents’ use of sanctions) were effective in achieving children’s recycling behavior, whereas environmental-specific parenting practices characterized by warmth (parents’ use of communication) were effective in achieving children’s reuse behaviors (for a discussion on environmental-specific parenting practices, see Katz-Gerro and colleagues, 2019) [41]. Similarly, among the few studies examining the influence of general parenting practices on pro-environmental outcomes, most of them have examined parental warmth rather than both parental warmth and strictness. General parenting practices characterized by warmth are positively related to pro-environmental outcomes [39,47]. For example, findings from a study [39] carried out in Denmark revealed that parental autonomy granting is positively related to adolescents’ motivation to engage in pro-environmental behavior. Although the relationship between parenting styles and socialization outcomes (e.g., internalization of social values) has been widely studied in the literature, few studies have examined the impact of parenting styles on environmental outcomes. Most of these seminal studies only used interviews, but not an orthogonal dimensional approach [47], or they examined empathy toward nature, but not internalization of environmental values [48].

Socialization in adolescence is a process characterized by an interaction between the individual and his/her relevant social contexts. Studies traditionally highlight parents as the main socialization agent in childhood, although in adolescence, apart from the family, other significant sources have a critical impact, such as peers and other informal sources like social media, television, or the Internet [3,49,50]. Several studies have shown that adolescents’ behavior depends on who these others are, as well as their degree of similarity in terms of age, attitudes, personality, and so on. Approval is more likely to be based on conformity with peer standards than with social norms [51]. Adolescence is a developmental time related to greater psychosocial vulnerability [27]. For example, some decrease in self-esteem might occur during adolescence. Similarly, in the social realm, although in adolescence there appears to be an increasing interest in environmental topics, adolescents report fewer pro-environmental behaviors than adults [39,40,52,53].

The present study aims to analyze the influence of parenting styles (i.e., authoritative, indulgent, authoritarian, and neglectful) on adolescent self-esteem and the priority given to environmental values. Based on previous studies, we expect that parenting styles characterized by higher levels of parental warmth (i.e., authoritative and indulgent styles) will be related to higher scores on self-esteem and internalization of environmental values than parenting styles characterized by lower levels of parental warmth (i.e., authoritarian and neglectful styles).

## 2. Materials and Methods

### 2.1. Sample and Procedure

In order to define the sample size, an a priori power analysis was conducted; the software used for the a priori power analysis was G*Power (Heinrich Heine University, Düsseldorf, Germany) [54,55]. Results showed that a minimum sample size of 304 observations was necessary to detect an unfavorable medium-small effect size (*f* = 0.24) with a power of 0.95 (α = 0.05, 1 − β = 0.95) on *F*-tests among the four styles [56,57,58]. The sample size obtained in the a priori power analysis was intentionally oversampled to provide sufficient observations to adequately address sensitivity. In the present study, participants were 308 Spanish adolescents with 171 females (55.5%) and 137 males (44.5%) between 12 and 17 years old (*M* = 14.59, *SD* = 1.4). According to the sensitivity statistical power analysis, for the sample size in the present study (*N* = 308), an effect size near 0.24 was estimated (*f* = 0.238, α = 0.05, 1 − β = 0.95). The research protocol was approved by the Research Ethics Committee of the Program for the Promotion of Scientific Research, Technological Development, and Innovation of the Spanish Valencian Region, the institution that supported this research. To achieve the planned sample, we contacted the heads of eight schools from the complete list of schools in a Spanish southern region. Parental approval and student consent forms were required. The questionnaires were administered during class time. All the participants in the present study were Spanish, as were their parents and four grandparents, and they lived in two-parent nuclear families with a mother or primary female caregiver and father or primary male caregiver. All of the assessments were completed anonymously. This study was also approved by the College Research Ethics Committee (CREC) of the Nottingham Trent University (NTU, Nottingham, UK; Project No. 2017/90).

### 2.2. Measures

#### 2.2.1. Parental Socialization

Parental warmth was captured with the warmth/affection scale (WAS) [59]. The WAS is composed of 20 items that measure the extent to which the adolescents perceive their parents as loving, responsive, and involved. The WAS scale is a commonly used instrument with good reliability and validity, and it has been used internationally on the five continents with more than 12,000 children [60]. Sample items are: “Make me feel what I do is important” and “Talk to me about our plans and listen to what I have to say”. Higher scores on the WAS scale represent higher levels of parental warmth. Cronbach’s alpha value was 0.938. Parental strictness was captured with the parental control scale (PCS) [59]. The PCS is composed of 13 items that measure the extent to which adolescents perceive strict parental control over their behavior. The PCS is a reliable and valid measure for cross-cultural research purposes, and it is widely used around the world, including in American ethnic groups [61]. Sample items are: “They believe in having a lot of rules and sticking to them” and “Tell me exactly what time I have to be home when I go out”). Higher scores on the PCS scale represent higher levels of parental strictness. Cronbach’s alpha was 0.791. On both scales, adolescents responded on a 5-point scale ranging from 1 (never) to 5 (always). Following previous research [17,18,21], the four parenting styles were defined using the median split procedure for parental warmth and parental strictness, examining the two parenting variables at the same time: Authoritative parenting (above the median on both warmth and strictness), indulgent parenting (above the median on warmth, but below on strictness), neglectful parenting (below the median on both variables), and authoritarian parenting (below the median on warmth, and above the median on strictness). It should be taken into account that the split procedure to assign families to the parenting groups, rather than assigning them on the basis of predetermined cutoffs, offers a categorization of families that is sample-specific. For example, families in the “authoritarian” group are indeed relatively more authoritarian (i.e., less warm and stricter) than the other families in the sample, although we do not know whether the families we have labeled “authoritarian” would be considered “authoritarian” within a different population. Thus, the designation of families as one type or another, in comparison to their counterparts, is done for heuristic, not diagnostic, purposes [17,32].

#### 2.2.2. Self-Esteem

Multidimensional self-esteem was measured with the AF5 scale (Garcia and Musitu, Madrid, Spain) [62]. The AF5 scale is composed of 30 items that measure self-esteem in five domains (i.e., academic, social, emotional, family, and physical). Academic dimension of self-esteem refers to the individual’s perception of the quality of the performance of their role as a student. Social dimension of self-esteem refers to the perception of their adaptation in social relationships, both for their ease of maintaining their social network and for some important qualities in interpersonal relationships. Emotional dimension of self-esteem refers to the perception of their general emotional state and their responses to specific relevant situations. Family dimension of self-esteem expresses the perception of their involvement, participation and integration in the family environment. Physical dimension of self-esteem indicates the perception of their appearance and physical condition, including their self-worth in sports practice. Sample items in the five domains are as follows: academic (“I do my homework well”), social (“I am a friendly person”), emotional (reverse scored, “I am afraid of some things”), family (“My family would help me with any type of problem”), and physical (“I take good care of my physical health”). The AF5 follows a multidimensional and hierarchical approach, based on Shavelson’s theoretical model [63]. The AF5 scale is a psychometrically sound questionnaire [64], developed and normed in Spain [62] and cross-culturally validated with Spanish-speaking samples from Chile [65], Portuguese-speaking samples from Portugal [66] and Brazil [64], and English-speaking samples from the United States [67]. The AF5 factorial structure of self-concept (multidimensional) was also confirmed in a non-western society (China) [68]. The Confirmatory Factor Analyses (CFA) provided evidence of the validity of the AF5 structure [67,69], without showing method effects related to negatively worded items [70]. Adolescents responded on a 99-point scale ranging from 1 (strong disagreement) to 99 (strong agreement). Modifications were made in order to obtain a score index ranging from 0.10 to 9.99. Higher scores in the five domains represent a greater sense of self-esteem. Cronbach’s alpha values were: academic, 0.764, social, 0.683, emotional, 0.672, family, 0.816, and physical, 0.770.

Global self-esteem was measured with the Rosenberg’s scale. It is composed of 10 items that measure overall feelings of self-worth or self-acceptance. A sample item is: “I feel that I have a number of good qualities”. Rosenberg’s scale, also validated in Spain [71], is one of the most widely used questionnaires in international research. Adolescents responded on a 4-point scale ranging from 1 (strongly disagree) to 4 (strongly agree). Higher scores indicate a greater sense of self-esteem. Cronbach’s alpha value was 0.823.

#### 2.2.3. Environmental Values

Adolescents’ environmental values were measured with six items that asked about the importance of environmental values as a guiding principle in their lives [2,72]. Sample items are: “Nature should not be polluted” and “Animals should not be mistreated”. Following some previous literature, environmental values were conceptualized as the so-called biospheric values. Specifically, biospheric values express the decision to act pro-environmentally, or not, based on the perceived costs and benefits for the ecosystem and biosphere as a whole (see de Groot and Steg, 2008, pp. 333–334) [73]. The importance given to biospheric values is positively associated with a broad spectrum of adjustment criteria in the environmental area, such as connectedness to nature, environmental concern, or ecological behaviors [74,75,76]. Adolescents responded on a 5-point scale ranging from 1 (never) to 5 (always). Higher scores indicate greater priority given to environmental values. Cronbach’s alpha value was 0.796.

### 2.3. Data Analysis

A multivariate factorial analysis of variance (MANOVA) was performed on self-esteem (academic, social, emotional, family, and physical) and environmental values. The factors included were parenting style (authoritative, authoritarian, indulgent, and neglectful), sex (males vs. females), and age (12–15 years old vs. 16–17 years-old) as independent variables. Follow-up univariate *F*-tests were applied for the multivariate sources that reached statistically significant differences, and significant results on the univariate *F*-tests were followed by Bonferroni comparisons of all possible pairs of means [21,77].

## 3. Results

### 3.1. Parenting Styles

Adolescent participants were classified into one of four parenting style groups (indulgent, authoritative, authoritarian, or neglectful; Table 1): authoritative parenting, with 76 participants (24.7%), based on higher warmth, *M* = 72.93, *SD* = 4.37, and higher strictness, *M* = 39.33, *SD* = 3.77; indulgent parenting, with 82 participants (26.6%), based on higher warmth, *M* = 72.51, *SD* = 4.30, and lower strictness, *M* = 28.22, *SD* = 4.59; authoritarian parenting, with 81 participants (26.3%), based on lower warmth, *M* = 54.07, *SD* = 11.00, and higher strictness, *M* = 39.60, *SD* = 4.99; and neglectful parenting, with 69 participants (22.4%), based on lower warmth, *M* = 53.55, *SD* = 11.57, and lower strictness, *M* = 29.09, *SD* = 3.80.

### 3.2. Multivariate Previous Analysis

Table 2 shows the results of the MANOVA. Significant differences were found in the main effects for parenting styles, Λ = 0.670, *F*(21, 821.8) = 5.855, *p* < 0.001, sex, Λ = 0.779, *F*(7, 286.0) = 11.564, *p* < 0.001, and age, Λ = 0.838, *F*(7, 286.0) = 7.879, *p* < 0.001. The results showed no statistically significant interaction effects (*p* > 0.005).

### 3.3. Parenting Styles, Self-Esteem, and Environmental Values

Table 3 shows the results of *F* values and Bonferroni test between parenting styles on self-esteem and environmental values. Overall, on multidimensional and global self-esteem, indulgent and authoritative parenting styles were associated with the optimal scores, whereas neglectful and authoritarian parenting styles were associated with poor scores. Specifically, on academic self-esteem, adolescents from indulgent families reported the highest scores, whereas the lowest scores corresponded to those from authoritarian and neglectful households. Along the same lines, adolescents who characterized their parents as indulgent and authoritative reported higher family self-esteem than those from non-warmth families (i.e., authoritarian and neglectful), with the lowest scores corresponding to authoritarian parenting. For physical self-esteem, a similar pattern was found, with higher scores for the indulgent and authoritative styles than for the authoritarian and neglectful styles, although differences between means only reached statistical level for the authoritative and neglectful styles. On global self-esteem, indulgent and authoritative parenting was related to higher scores than authoritarian and neglectful parenting. On the other hand, a similar tendency to that of the self-esteem outcomes was found for environmental values. Adolescents who characterized their parents as indulgent and authoritative gave the greatest priority to environmental values, whereas their peers from authoritarian and neglectful households had the lowest scores.

### 3.4. Sex- and Age-Related Differences in Self-Esteem and Environmental Values

Table 4 shows sex- and age-related differences in self-esteem and environmental values. Sex-related differences indicated that males obtained higher scores than females on emotional and physical self-esteem, whereas females obtained higher scores than males on the internalization of environmental values. Age-related differences indicated that, in turn, adolescents from 12 to 15 years old obtained higher scores than those from 16 to 18 years old on family self-esteem, whereas adolescents aged 16 to 18 obtained higher scores than those aged 12 to 15 on internalization of environmental values.

## 4. Discussion

The aim of this study was to further assess the effects of parenting styles (authoritative, indulgent, authoritarian, and neglectful) on Spanish adolescents’ self-esteem and pro-environmental values. Self-esteem was evaluated in five spheres: academic, social, emotional, family, and physical. Additionally, a global measure of self-esteem was considered. Pro-environmental values were evaluated as the importance of protecting nature as a guiding principle in adolescents’ lives. Importantly, results showed a common pattern between parenting styles and their contribution to an adequate sense of self (i.e., self-esteem), as well as a sense of being part of the natural environment (i.e., environmental values). Overall, findings from the present study showed that parenting styles characterized by high levels of affection and dialogue, the indulgent and authoritative styles, were related to the best self-esteem outcomes. Regarding the internalization of environmental values, the results showed that higher levels of this internalization were associated with authoritative and indulgent parenting styles.

Specifically, the relationship between parenting styles and self-esteem and pro-environmental values showed a consistent pattern. Adolescents from families characterized by warmth (i.e., indulgent and authoritative) reported greater self-esteem in academic, social, and emotional spheres than those from non-warmth families, although on academic self-esteem, the authoritative group did not reach a statistically significant level, whereas on physical self-esteem, the indulgent group did not reach a statistically significant level. A similar tendency was found for global self-esteem. Adolescents who characterized their parents as indulgent or authoritative reported more global self-esteem than their counterparts from authoritarian and neglectful families. On environmental values, again, only adolescents from families characterized by warmth reported giving a high priority to environmental values, whereas the poorest internalization of environmental values corresponded to their peers from authoritarian and neglectful families. The findings from this study agree with some previous studies, mainly carried out in European and Latin-American countries, about the positive impact of parental warmth and involvement on adolescent self-esteem [21,27,34,78] and priority given to social values [7,36,79,80]. Although these studies examined the contribution of parenting styles to self-esteem and internalization of social values, the present findings offer new and crucial empirical evidence in the less explored domain of the environmental field, extending the evidence about the impact of parenting styles from social values (self-transcendence and conservation values) to environmental values, an essential but less studied domain in the area of parental socialization.

Parents have a crucial impact on children and adolescents’ development of self-esteem and internalization of social values. As Grusec and Goodnow (1994) highlight [5], for the internalization of values, it is necessary to take into consideration aspects of children and adolescents’ psychological adjustment, such as self-esteem. Lower levels of self-esteem could obstruct values internalization. Within social values, self-transcendence and conservation values are widely considered socially focused values [81,82,83] and theoretical constructs centered on consideration for others and agreement with social norms are positively related to interpersonal empathy [81,84,85,86,87]. Nevertheless, the internalization of environmental values as an adolescent outcome variable has not been purposely examined, even though different scholars have stressed the importance of parental socialization in the internalization of social values, including environmental values—commonly operationalized as biospheric values. In general, previous parenting research has been limited to isolated pro-environmental outcomes [39,47,48], without considering individual aspects of the child (e.g., self-esteem) in the outcome criteria.

It is important to know, therefore, what parents can do to achieve well-adjusted children oriented toward nature conservation, although previous environmental research shows discrepancies in explaining how parents respond to environmental socialization by applying environmental-specific parenting practices. For example, parents can insist that their child turn off lights to save electricity, withdrawing privileges due to noncompliance—a parenting practice characterized by strictness. Some inconsistent findings revealed a differential effectiveness of parenting strategies depending on the pro-environmental outcomes [45]. Environmental-specific parenting practices characterized by strictness (parents’ use of sanctions) are effective in achieving children’s recycling behavior, but environmental-specific parenting practices characterized by warmth (parents’ use of communication) are effective in achieving children’s reuse behaviors. However, other pro-environmental scholars [41] did not find any relationship between specific strict parenting practices and children’s environmental behavior (for a discussion, see Katz-Gerro and colleagues, 2019) [41]. In contrast, findings from the present study revealed that the impact of the parenting style shows the same pattern for adolescents’ self-esteem and their internalization of environmental values; that is, only adolescents from families characterized by warmth have greater self-esteem and give a higher priority to environmental values.

Overall, the present study suggests that both authoritative and indulgent parenting styles have a positive effect on psychosocial outcomes (self-esteem and environmental values internalization) in Spanish adolescents. Furthermore, in cultural contexts such as European and Latin-American countries, different studies have shown that the indulgent parenting style has been related to optimal outcomes in children and adolescents’ psychosocial adjustment [21,26,31,34,36,80,88]. Indulgent parenting has a positive impact on children’s personal and social adjustment [89], offering protection against alcohol use and abuse, promoting motivations for drinking and non-drinking [90], or preventing personal maladjustment [91]. However, these empirical findings also contrast with some studies conducted with families from other cultural contexts; it is important to note that the parenting literature has shown cultural variations in the optimal style [6,19,80,88,92]. In particular, empirical research from European-American contexts revealed that the best parenting is based on parental strictness accompanied by parental warmth [6,15,17], and even parental strictness without parental warmth emerges as a good parenting strategy in ethnic minorities from the United States or families from Arabic and non-western countries [23,25,93,94].

Although it is not central to this study, our results also confirm some sex- and age-related differences found in other previous studies with adolescents. Regarding age-related differences, early adolescents tend to report greater family self-esteem than late adolescents, whereas late adolescents give more priority to environmental values than early adolescents. Regarding sex-related differences, males report higher emotional and physical self-esteem than females. These results agree with some important studies in the area of adolescence [27,88,91].

The present study and its findings should be interpreted taking some limitations into account. The study has a cross-sectional design, and so we cannot draw conclusions about the directionality and causal relationships between the variables. It should be noted that, in the absence of longitudinal data, the findings from this study should be considered preliminary [21]. Additionally, parenting was captured through parental measures provided by adolescent children rather than their parents, although similar results have been obtained with measures provided by other sources, such as external informants or reports from parents [17,18]. Despite these drawbacks, the present study offers new and interesting evidence about how parents can contribute to the environmental socialization of their adolescent children. Moreover, the use of the theoretical framework based on the dimensional model with four parenting styles also encourages future studies to further examine parenting styles and other environmental outcomes.

## 5. Conclusions

The present study provides new evidence about the crucial role of parents in adolescent self-esteem and internalization of environmental values. Previous research on environmental socialization offers inconsistent findings about which environmental-specific parenting practices would be most appropriate in environmental socialization [41]. By contrast, the present study revealed a consistent pattern between parenting styles and adolescent self-esteem and internalization of environmental values. It is important to note that differences in self-esteem and internalization of environmental values in adolescents can be predicted by parenting styles. Only parents who use warmth, reasoning, and involvement—regardless of their levels of parental strictness—have well-adjusted adolescents who have higher levels of self-esteem and give greater priority to environmental values. These findings clearly contrast with those obtained in other cultural contexts where parental strictness is an essential component in achieving well-adjusted children with optimal psychosocial development. Therefore, the cultural context where parental socialization takes place seems to be essential in explaining the impact of parental warmth and strictness on adolescent development.

## Figures and Tables

**Table 1:** Distribution of families according to parenting style, means (*M*), and standard deviations (*SD*) on parental warmth and strictness.

	Total	Authoritative	Indulgent	Authoritarian	Neglectful
Frequency	308	76	82	81	69
Percent	100.0	24.7	26.6	26.3	22.4
Warmth					
Mean	63.52	72.93	72.51	54.07	53.55
SD	12.66	4.37	4.30	11.00	11.57
Strictness					
Mean	34.15	39.33	28.22	39.60	29.09
SD	6.96	3.77	4.59	4.99	3.80

**Table 2:** Factorial MANOVA (4 ^a^ × 2 ^b^ × 3 ^c^) of self-concept, self-esteem, and ecological socialization.

Source of Variation	Λ	*F*	*df*_between_	*df*_error_	*p*
(A) Parenting Styles ^a^	0.670	5.855	21.0	821.8	<0.001
(B) Sex ^b^	0.779	11.564	7.0	286.0	<0.001
(C) Age ^c^	0.838	7.879	7.0	286.0	<0.001
A × B	0.925	1.074	21.0	821.8	0.371
A × C	0.921	1.144	21.0	821.8	0.295
B × C	0.982	0.763	7.0	286.0	0.619
A × B × C	0.932	0.971	21.0	821.8	0.497

^a^*a*_1_, authoritative, *a*_2_, indulgent, *a*_3_, authoritarian, *a*_4_, neglectful; ^b^*b*_1_, male, *b*_2_, female; ^c^*c*_1_, adolescents (12–15 years), *c*_2_, adolescents (16–18 years).

**Table 3:** Means, standard deviations (in parenthesis), *F* ^#^ values, and Bonferroni test between parenting styles on self-esteem and environmental values.

	Authoritative	Indulgent	Authoritarian	Neglectful	*F* (3, 292)
Multidimensional Self-Esteem					
Academic	6.49	7.14 ^1^	6.23 ^2^	5.88 ^2^	8.295 ***
	(1.85)	(1.75)	(1.57)	(1.83)	
Social	7.48	7.56	7.30	7.18	0.637
	(1.65)	(1.57)	(1.50)	(1.45)	
Emotional	5.51	5.55	5.61	5.52	0.217
	(1.96)	(1.81)	(2.12)	(1.94)	
Family	8.84 ^1^	9.14 ^1^	6.95 ^3^	7.67 ^2^	33.397 ***
	(1.04)	(0.77)	(2.21)	(2.00)	
Physical	6.71 ^1^	6.43	5.91	5.82 ^2^	3.475 *
	(1.85)	(2.12)	(2.06)	(2.05)	
Global Self-Esteem	33.07 ^1^	32.60 ^1^	29.86 ^2^	30.14 ^2^	7.040 ***
	(4.54)	(5.75)	(5.59)	(5.09)	
Environmental Values	3.45 ^1^	3.25 ^1^	2.98 ^2^	2.97 ^2^	3.517 *
	(0.95)	(1.05)	(0.96)	(1.02)	

^#^ α = 0.05; * *p* < 0.05; *** *p* < 0.00, ^1^ > ^2^ > ^3^.

**Table 4:** Means, standard deviations (in parenthesis), *F* ^#^ values for sex and age on self-esteem and environmental values.

	Sex			Age		
	Female	Male	*F* (1, 308)	12–15 Years	16–18 Years	*F* (1, 308)
Multidimensional Self-Esteem						
Academic	6.62	6.27	3.013	6.57	6.34	3.364
	(1.79)	(1.80)		(1.84)	(1.75)	
Social	7.44	7.31	0.886	7.51	7.25	1.966
	(1.48)	(1.62)		(1.51)	(1.57)	
Emotional	5.11	6.09	18.651 ***	5.62	5.48	0.054
	(1.91)	(1.87)		(2.06)	(1.83)	
Family	8.10	8.24	0.095	8.68	7.60	38.732 ***
	(2.02)	(1.59)		(1.54)	(1.97)	
Physical	5.69	6.90	27.742 ***	6.24	6.20	0.001
	(1.96)	(1.96)		(2.05)	(2.05)	
Global Self-Esteem	30.94	32.08	2.505	32.07	30.77	3.617
	(5.34)	(5.52)		(5.30)	(5.54)	
Environmental Values	3.36	3.01	7.940 ***	2.92	3.40	15.877 ***
	(0.99)	(1.00)		(1.00)	(0.96)	

^#^ α = 0.05; *** *p* < 0.001.
